# Prolonged Somatostatin Receptor 2 Antagonism Enhances Glucagon Response to Hypoglycemia in Male Diabetic Rats

**DOI:** 10.1210/endocr/bqaf192

**Published:** 2026-01-08

**Authors:** Ninoschka C D’Souza, Nadia Aleali, Dorsa Shakeri, Sara C Atherley, Emily G Hoffman, Sina Karimi Chahartash, Sahel Javanbakhsh, Owen Chan, Richard T Liggins, Michael C Riddell

**Affiliations:** School of Kinesiology & Health Science, York University, Toronto, ON M3J 1P3, Canada; School of Kinesiology & Health Science, York University, Toronto, ON M3J 1P3, Canada; School of Kinesiology & Health Science, York University, Toronto, ON M3J 1P3, Canada; School of Kinesiology & Health Science, York University, Toronto, ON M3J 1P3, Canada; School of Kinesiology & Health Science, York University, Toronto, ON M3J 1P3, Canada; School of Kinesiology & Health Science, York University, Toronto, ON M3J 1P3, Canada; School of Kinesiology & Health Science, York University, Toronto, ON M3J 1P3, Canada; Department of Internal Medicine, University of Utah, Salt Lake City, UT 84112, USA; Zucara Therapeutics Inc, Vancouver, BC V6T 1Z3, Canada; School of Kinesiology & Health Science, York University, Toronto, ON M3J 1P3, Canada

**Keywords:** glucagon, SSTR2a, hypoglycemia, type 2 diabetes, counterregulation, somatostatin

## Abstract

In diabetes, glucagon is typically oversecreted during hyperglycemia but undersecreted during hypoglycemia. Administration of a somatostatin receptor antagonist (SSTR2a) increases glucagon counterregulation during hypoglycemia in rodent models of type 1 diabetes (T1D) but less is known about its effect on glucagon in type 2 diabetes (T2D). Using a rodent model of insulin-requiring diabetes, we evaluated the effects of daily SSTR2a administration with insulin dosing (study A: 8 days) and repeated exposures to hypoglycemia (study B: 4× over 11 days) on glucagon and glycemia. In study A, 8 days of SSTR2a treatment at 3.0 mg/kg transiently increased glucagon levels after dosing but did not significantly affect the glycemic response to basal or bolus insulin. In study B, with daily low-dose SSTR2a treatment (0.3 mg/kg/d), the glucagon counterregulatory response to insulin-induced hypoglycemia increased while time to hypoglycemic onset was delayed on challenge days 1 and 2. SSTR2a treatment did not affect food intake, body mass, or C-peptide levels, but was associated with a lower glycated hemoglobin A_1c_ level at the end of the study relative to controls (4.3 ± 0.9 vs 5.3 ± 0.8%; *P* < .05). In summary, in a rat model of insulin-treated T2D, daily SSTR2a administration increased glucagon counterregulation to hypoglycemia without worsening overall insulin sensitivity or glycemic control.

Hypoglycemia is a common and potentially hazardous complication of glucose-lowering therapies in diabetes. Almost all individuals with type 1 diabetes (T1D) and approximately 40% of individuals with type 2 diabetes (T2D) experience hypoglycemia at various frequencies depending on the therapies they employ for glycemic control, with exogenous insulin therapy being associated with the highest risk for hypoglycemic exposure (1). Hypoglycemia is categorized as level 1 (3.0-3.9 mmol/L) or level 2 (<3.0 mmol/L), or as severe hypoglycemia (SH) if assistance is required for recovery, regardless of the prevailing blood glucose concentration. Among those with T2D, SH is primarily observed in individuals treated with sulfonylureas or insulin therapy (1, 2) and the combined rate of level 2 (<3.0 mmol/L) and level 3 events may be as high as 7.10 events per person-year in individuals treated with basal-only insulin and double that for those employing basal-bolus insulin therapy (3). Furthermore, intensification of the insulin regimen increases the risk for SH (4-7), which can be linked to increased cardiovascular mortality and morbidity risk (8).

Hypoglycemia occurs not only due to excess insulin exposure, but also because of a failure of the counterregulatory processes that normally regulate falling blood glucose levels. In individuals without diabetes, a decline in endogenous insulin secretion (from β cells) and a rise in glucagon secretion (from α cells) limit hypoglycemia when blood glucose levels decline to approximately 3.5 mmol/L. Both physiologic responses can become blunted, or lost, in T1D and T2D, with blunting exacerbated by prior hypoglycemia exposure (9-11). Repeated (antecedent) hypoglycemic events put individuals at further risk for another more severe event (12, 13) and can also result in loss of symptom awareness (14), further increasing the risk for SH (15). Intensification in glycemic management in T1D and T2D has become a goal of disease management, resulting in decreased hyperglycemia; however, this has also coincided with an increased prevalence of all levels of hypoglycemia (16).

The insulin and glucagon responses are linked by electrical coupling of the pancreatic α and β cells (17), and by the intraislet control of glucagon secretion by insulin, involving somatostatin (SST) secreted by δ cells, which suppresses glucagon secretion via SST receptor 2 (SSTR2) on the α cell (18). Accordingly, it has been hypothesized that the blunted counterregulatory response in diabetes results, at least in part, from impaired intracellular communication within the islet, including excess SST signaling, which suppresses glucagon release (19), and that SSTR2 antagonist (SSTR2a) administration can ameliorate this imbalance (20-22).

Beyond its effects on the counterregulatory response to hypoglycemia, glucagon dysregulation in T2D may have other effects, including hyperglucagonemia, which can worsen blood glucose control. Small increases in α-cell glucagon secretion normally occur at mealtimes, which augment insulin secretion from surrounding β cells (23); however, excess postprandial glucagon secretion in T2D can worsen hyperglycemia by increasing hepatic glucose production (24). Glucagon also has regulatory roles on lipid and amino acid metabolism, mobilizing both substrates for energy metabolism in the fasted and exercising states (25, 26). Small elevations in circulating glucagon levels may also potentiate weight loss by promoting satiety, increasing energy expenditure, and by promoting lipolysis in the adipose tissue and liver (27).

Paradoxically, both glucagon receptor antagonists (28, 29) and agonists, combined with incretin therapy to boost insulin secretion (30), have shown efficacy for the treatment of obesity and T2D (27, 31, 32).

Augmentation of glucagon secretion, with administration of amino acids (33, 34) or SSTR2a (21) in T2D, may have substantial metabolic implications for individuals living with T2D. Impaired counterregulation of hypoglycemia may be improved (21) while body weight management could also be affected (35). On the other hand, increases in endogenous glucagon secretion, or increases in systemic levels from glucagon administration, may counteract the action of exogenous insulin treatment and deteriorate overall glycemic control (36). While a SSTR2a is being evaluated as an approach to restoring glucagon counterregulatory failure in rodent models of diabetes (37), other potential effects of acute and chronic SSTR2a administration on glucose metabolism in T2D on are not known.

The purpose of this study was to assess the effects of daily SSTR2a administration for up to 11 days on overall glycemia and glucagon responses, in a male rat model of insulin-treated T2D, with and without hypoglycemia. We hypothesize that prolonged SSTR2 antagonism will not exacerbate glycemia and hormone responses in animals with diabetes.

## Materials and Methods

### Type 2 Diabetes Animal Model

Animal studies were conducted at York University following ethics approval by the university's animal care committee and in accordance with Canadian Council for Animal Care guidelines. Male Sprague Dawley rats, obtained at age 8 weeks (strain 001, Charles River Laboratories) were housed at 23 to 25 °C, with a 12-hour light/dark cycle and ad libitum access to food and water. Rats were fed a high-fat diet for 3 weeks prior to diabetes induction (60% fat, 20% carbohydrates, 20% protein, ResearchDiets; catalog No. D12492) and for the remainder of the protocol, to induce baseline obesity and insulin resistance, as previously described (21). Diabetes was induced at approximately age 11 weeks, with a single intraperitoneal injection of 35 mg/kg streptozotocin (STZ), to achieve a hyperglycemia target of 13.9 mmol/L (morning basal blood glucose level). Twenty-four hours after STZ injection (day 1), diabetic rats began receiving daily titrated insulin maintenance doses, between 1 and 3 U/day, depending on daily blood glucose as previously described (21). Rats were monitored daily for food intake, body weight, and morning (fed) and evening (postabsorptive) blood glucose using a glucometer (Contour Next Ascensia Diabetes Care). Blood ketone levels were monitored daily (Freestyle Precision Neo meter, Abbott Diabetes Canada). On day 8, rats were randomly assigned to treatment groups.

#### Hormone measurements

For determination of glucagon and C-peptide levels, blood samples were obtained prior to SSTR2a or vehicle administration, and during insulin challenges, 60 minutes and immediately prior to insulin bolus dosing, and every 30 minutes for 120 minutes thereafter. Blood samples (∼200 µL) were collected from the saphenous vein using a 25-gauge needle into EDTA-coated tubes (catalog No. 16.444.100, Starstedt), then centrifuged (12 000 rpm for 5 minutes) and the plasma separated and stored at −80 °C prior to analysis. Enzyme-linked immunosorbent assays (ELISAs) were used for glucagon (Mercodia catalog No. 10-1271-01, RRID: AB_2737304), C-peptide (Crystal Chem catalog No. 90055, RRID: AB_2893130) measurement. Conclusions on glucagon responses were calculated in terms of the total glucagon, basal-peak glucagon as well as the time to the maximal glucagon response (Tmax) and has been illustrated accordingly. Glycated hemoglobin A_1c_ (HbA_1c_) was determined (Crystal Chem catalog No. 80300) as previously described (21).

#### Study A: effects of daily somatostatin receptor antagonist administration without hypoglycemia

**Figure 1.**
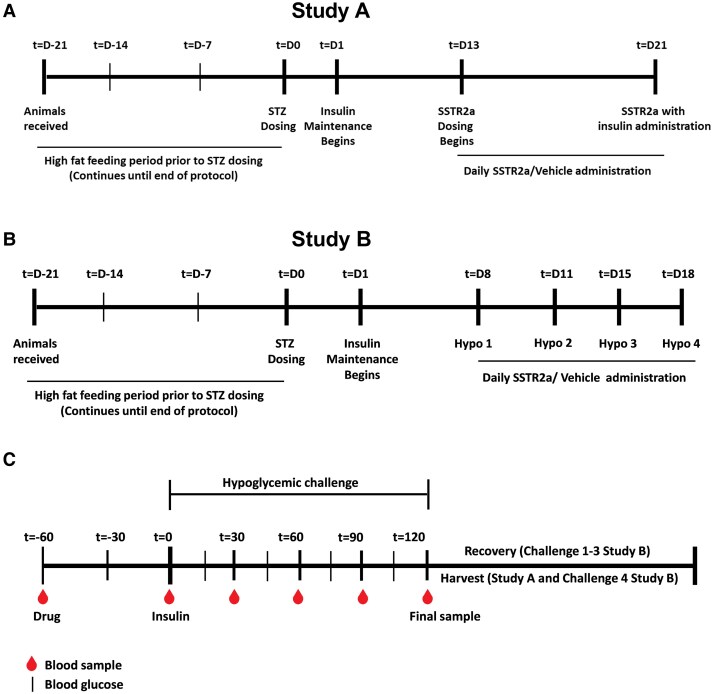
Schematic representation of study protocols and insulin-induced hypoglycemic challenges. Study A and B involved daily dosing of A, somatostatin receptor antagonist (SSTR2a) or vehicle with a mild insulin exposure or B, multiple hypoglycemic challenges, respectively. Rats were administered streptozotocin (STZ) on day 0 to induce diabetes for studies A and B, respectively. The period of daily SSTR2a (or vehicle) administration is indicated for each study. Each challenge involved drug dosage at t = −60 followed by insulin administration at t = 0. Animals were then rescued after t = 120 (challenges 1-3 of study B) or harvested (study A and challenge 4 of study B). C, Blood sample collection and blood glucose readings were taken at regular intervals as indicated by the red blood droplets and black lines, respectively.

Beginning on day 13, rats received daily 3-mg/kg/d SSTR2a (or vehicle) administration (provided by Zucara Therapeutics Inc) for 8 days until day 21. The 3-mg/kg daily dose level was selected to assess the effect of daily SSTR2a exposure, as this dose level of SSTR2a was previously shown to be at the high end of the drug's effective dose range in preventing hypoglycemia in T1D rats (22). After 8 daily SSTR2a doses, on day 21, rats received 1.2-U/kg bolus insulin aspart in the postprandial state (late morning) to assess their blood glucose response to insulin. This dose level for insulin was selected to test exogenous (ie, therapeutic) insulin effectiveness by mimicking bolus insulin requirements to normalize hyperglycemia with meals. This allowed for an assessment of the effect of repeated SSTR2a administration on glycemia with exogenous insulin therapy. Plasma glucagon and C-peptide levels were assessed on days 1, 5, and 8 of SSTR2a administration, immediately prior to and 1 hour after dosing. The timeline for study A is summarized in Fig. 1A.

#### Study B: effects of daily somatostatin receptor antagonist administration with repeated hypoglycemia challenges

Beginning on day 8, after initiating bolus insulin maintenance dosing, SSTR2a-naive T2D rats received daily 0.3-mg/kg/d SSTR2 (or vehicle) administration for 11 days until day 18. The 0.3-mg/kg daily dose level was selected to assess the effect in hypoglycemia challenges as it was previously shown to be the minimally effective dose in reducing hypoglycemia exposure in T1D (22). During this period, rats underwent 4 insulin-induced hypoglycemic challenges (day 8, 11, 15, and 18), each separated by 2 or 3 days. The timeline for study B is summarized in Fig. 1B.

#### Hypoglycemia challenges

In study B, rats were administered SSTR2a or vehicle 1 hour prior to bolus insulin treatment (12-U/kg insulin aspart for challenges 1 to 3 and 6 U/kg insulin aspart for challenge 4). The insulin dosage was lowered for the final challenge to account for the effects of antecedent hypoglycemia. Blood glucose was measured every 15 minutes using a glucometer (see Fig. 1C), and plasma for hormone measurements were collected every 30 minutes as previously described (21). Two hours after insulin treatment, rats were treated with a 35% dextrose solution for recovery from hypoglycemia, or earlier, as a rescue intervention, if glucose levels dropped below 1.4 mmol/L. Dextrose (2 mL) was administered initially by intraperitoneal (IP) injection with monitoring every 15 minutes and additional dextrose by IP injection or oral gavage, as required, if blood glucose did not rise more than 3.9 mmol/L in response to the first dextrose dose. No insulin maintenance was provided the evenings of the hypoglycemic challenges to allow the animals to recover sufficiently and prevent a relapse in hypoglycemia, caused by sustained insulin action and/or deficient counterregulation.

### Statistical Analysis

Statistical analysis was completed using GraphPad PRISM (version 10) software. Treatment effects over time were assessed by analysis of variance with Tukey post hoc tests as appropriate. Unpaired *t* tests were performed with Welsh's correction. Rates and times of onset of hypoglycemia were compared by a Mantel-Cox test of survival curves. Statistical significance was defined by *P* less than .05.

### Outlier Adjustments

The mean fold-change in glucagon following the day 8 insulin challenge in study A was calculated after excluding 2 values in the SSTR2a group that demonstrated even greater drug effect (48-fold and 101-fold increase), since they were statistical outliers and would bias the mean values toward an even greater response with SSTR2a. No exclusions were made for daily monitoring analysis.

## Results

### Study A: Daily Somatostatin Receptor Antagonist Administration Did Not Worsen Glycemia or Exogenous Insulin Effectiveness

After STZ injection, all rats became hyperglycemic and by day 4, mean morning glucose levels were 24.1 ± 4.1 and 26.2 ± 5.4 mmol/L in the rats subsequently randomly assigned to the vehicle and SSTR2a groups, respectively (*P* = .40). Blood glucose remained consistently in the hyperglycemic range (>10.0 mmol/L), and rats received a mean 3.7-U/kg/d insulin for maintenance of blood glucose levels throughout the remainder of the study. N = 8 rats were assigned to each group (vehicle or SSTR2a) on day 13, prior to beginning daily SSTR2a administration.

**Figure 2.**
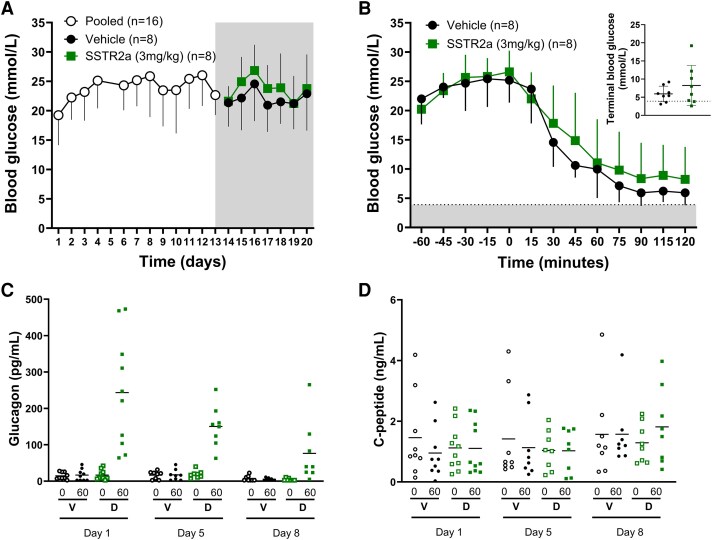
Blood glucose and hormone responses to daily somatostatin receptor antagonist (SSTR2a) administered at baseline (hyperglycemic) conditions. A, Daily morning glucose levels of all animals before and after daily SSTR2a administration. The shaded gray region denotes the daily dosing period. Panel B denotes blood glucose response with SSTR2a administration at t = −60 and insulin (1.2 U/kg) at t = 0 with the hypoglycemic zone denoted by the shaded gray region. Terminal glucose levels (B inset) were noted at t = 120 with a hypoglycemic threshold of 3.9 denoted by a dotted line. Hormone analysis for C, glucagon and D, C-peptide is provided in scatter plots for time points immediately prior to (0) and 1 hour after (60) SSTR2a or vehicle administration on days 1, 5, and 8. All animals are denoted by open (baseline) or closed (posttreatment) symbols for SSTR2a- (squares) and vehicle- (circles) treated groups. Data are presented as mean ± SD for line graphs. The center line in panels C and D denote the mean. D, SSTR2a; V, vehicle; N = 8 for all groups.

Daily SSTR2a administration for 8 days did not affect basal glucose levels (Fig. 2A), body weight changes, or food intake (data not shown). The 1.2-U/kg insulin dose was one-third of the mean daily insulin maintenance dosage and lower than doses previously reported to induce hypoglycemia in this model (6-12 U/kg) (21). Furthermore, after the 8 daily SSTR2a doses, there was no effect of SSTR2a on the blood glucose response to a 1.2-U/kg bolus dose of rapid-acting insulin (Fig. 2B). Following insulin dosing, one rat (in the SSTR2a group), although hyperglycemic, did not respond to insulin administration, with no change in blood glucose after treatment, and was excluded from subsequent analysis. Among the remaining rats, blood glucose declined from baseline glucose levels of 22.0 ± 4.4 mmol/L in the vehicle group and 19.8 ± 2.1 mmol/L in the SSTR2a group to a mean nadir of 5.4 ± 1.7 and 7.3 ± 5.5 mmol/L in the vehicle and SSTR2a groups, respectively (*P* = .4). Two rats in the vehicle group and 3 rats in the SSTR2a group experienced hypoglycemia (<3.9 mmol/L), with onset between 60 and 115 minutes; the remainder achieved and maintained euglycemia (4-10 mmol/L). Therefore, repeated SSTR2a administration did not adversely affect the glycemic response to therapeutic insulin. Basal glucagon levels (measured at t = 0, prior to daily dosing) declined approximately 2-fold over the 8-day dosing period in both groups (effect of time; *P* = .0042), from 14.5 ± 9.5 to 7.4 ±7.5 pg/mL on dosing days 1 and 8 in the vehicle group (experimental days 13 and 21), and close to 4-fold from 16.7 ± 13.5 to 4.3 ± 3.3 pg/mL in the SSTR2a group (Fig. 2C), but in a treatment-independent manner (*P* = .89). To account for the change in baseline glucagon over time, this measure was also evaluated as a fold-change from baseline. Glucagon levels increased 1 hour after SSTR2a administration on all days (1, 5, and 8). Although the absolute increase in glucagon declined from day 1 to 8 in a similar manner in both groups, the average fold-change on all days was much higher in the SSTR2a group (10- to 13-fold increase) compared to the vehicle group (1.5-fold). This treatment effect (*P* < .0001) was independent of time (*P* = .70). C-peptide levels were not affected by SSTR2a administration over the 8 dosing days. Similar levels were observed on days 1, 5, and 8, both pre and post dose for both groups (Fig. 2D).

Overall, the findings in study A demonstrate that repeated exposure to SSTR2a at a high dose did not worsen glycemia, glucagon responses, or C-peptide levels. While one of the goals of this study was to assess the possible effect of SSTR2 antagonism on the efficacy of bolus insulin to restore euglycemia, the dose given may have been too high as some of the animals developed hypoglycemia in both treatment groups. Thus, the responsiveness to insulin was further investigated in study B with repeated exposure to hypoglycemic challenges, while treated with prolonged drug exposure, but at a lower dose level.

### Study B: No Adverse Effects on Glycemia and Hormone Responses With Daily Somatostatin Receptor Antagonist Administration and Repeated Hypoglycemic Challenges

#### Improved glycemic outcomes with somatostatin receptor antagonist exposure

**Figure 3.**
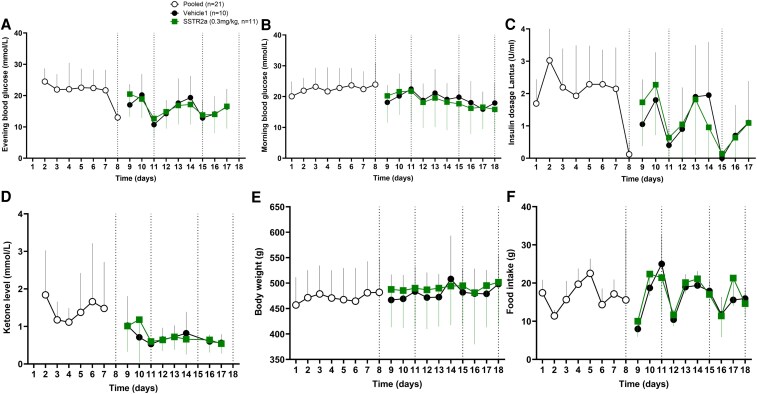
Daily monitoring data for study B. Daily blood glucose data was recorded A, in the evening and B, morning for all animals in study B post diabetes induction (day 1). Somatostatin receptor antagonist (SSTR2a) or vehicle dosing was initiated on day 8 and continued until the end of the protocol. Blood glucose data were pooled prior to treatment (indicated by open white circles) and then plotted separately for SSTR2a (green squares) and vehicle (black circles) groups. Hypoglycemic challenges were conducted on days 8, 11, 15, and 18 (indicated by the vertical dotted line) in all graphs. C, Daily evening insulin levels; D, ketones; E, body weight; and F, food intake are also shown. Data are presented as mean ± SD for line graphs.

**Figure 4.**
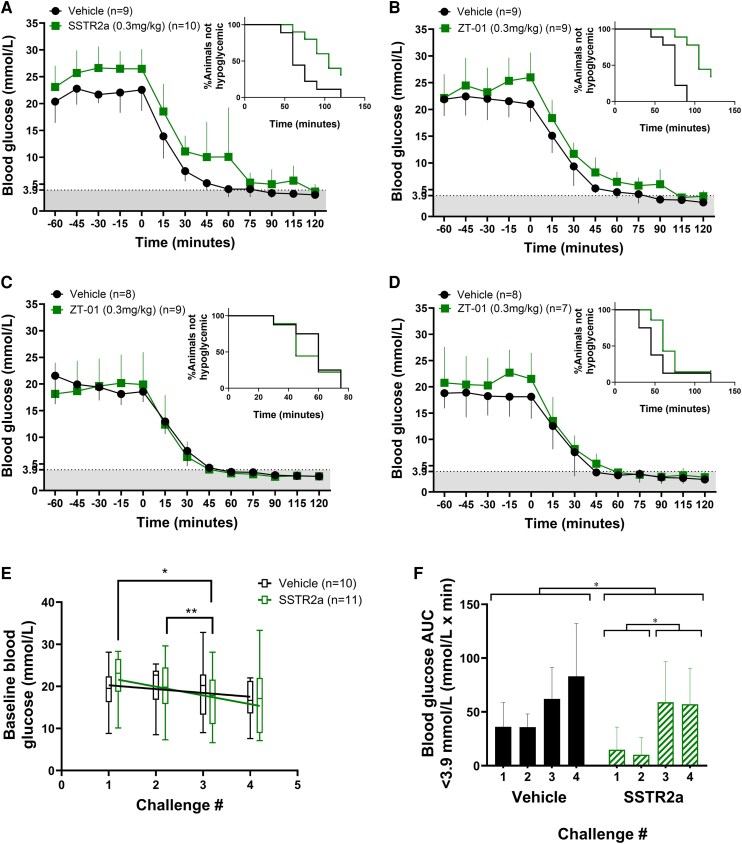
Blood glucose response to 4 successive insulin-induced hypoglycemic challenges. Blood glucose responses over the 4 hypoglycemic challenges (A-D, respectively) along with percentage of animals not hypoglycemic (A-D insets) were recorded for each hypoglycemic challenge. The somatostatin receptor antagonist (SSTR2a) group is indicated by green squares and vehicle with black circles. Linear regression analysis was conducted for baseline (prechallenge) blood glucose levels in hypoglycemic challenges 1 to 4 and mean levels represented as E, box-whisker plots (center line indicates median; box represents interquartile range; whiskers extend from maximum to minimum values) and F, blood glucose area under the curve less than 3.9 mmol/L after challenge 4 are also provided. The gray shaded region denotes the hypoglycemic zone (<3.9 mmol/L). All data are presented as mean ± SD and E and F, statistical significance is denoted by *P less than .05 and **P less than .01 in challenges 1 to 4.

After STZ injection, all rats became hyperglycemic and on day 3, morning glucose levels were comparable, with means of 22.5 ± 3.4 and 23.2 ± 7.7 mmol/L (Fig. 3B), in the rats subsequently randomly assigned to the vehicle and SSTR2a groups, respectively (*P* = .80), and morning and evening (Fig. 3A) blood glucose remained consistently in the hyperglycemic range (>10.0 mmol/L) until the first hypoglycemic challenge on day 8. Following this, morning (fed) glucose levels declined significantly from day 8 to 18 at mean rates of 0.4 ± 0.2 and 0.7 ± 0.1 mmol/L/day in the vehicle and SSTR2a groups, respectively (*P* = .026 for vehicle, and *P* < .0001 for SSTR2a; Fig. 3B), which is also reflected a decline in the insulin dosages required (Fig. 3C). With antecedent hypoglycemia, mean baseline blood glucose level prior to each challenge also declined (Fig. 4E) at a constant rate by 0.9 and 2.6 mmol/L per challenge for vehicle- and SSTR2a-treated groups, respectively, which was statistically significant for SSTR2a-treated rats (*P* = .02; *R^2^* = 0.96) but not vehicle (*P* = .27; *R^2^* = 0.53). On day 18, mean basal glucose was 17.9 ± 6.6 and 15.9 ± 7.8 mmol/L in the vehicle and SSTR2a groups, respectively. By day 18, 20% of vehicle- and 40% of SSTR2a-treated rats were no longer hyperglycemic, with baseline blood glucose values prior to insulin challenge below 10.0 mmol/L. Mean HbA_1c_ values, determined on day 18, were 5.3 ± 0.9 vs 4.3 ±0.9 for vehicle-and SSTR2a-treated rats (*P* = .04). All rats meeting the more than 10.0 mmol/L threshold for hyperglycemia at baseline on challenge days were included in the evaluation of effects during insulin-induced hypoglycemia, which included n = 9 vehicle-treated rats for challenges 1 and 2 and n = 8 rats for challenges 3 and 4 and for SSTR2a-treated rats, n = 10 in challenge 1, n = 9 in challenge 2 and 3, and n = 7 in challenge 4. SSTR2a had no effect on blood ketones, body weight, or food intake (Fig. 3D-3F). The mean maintenance insulin daily dose for all rats in study B was 3.4 U/kg.

Across the hypoglycemic challenges, a statistically significant effect of SSTR2a was observed: delayed onset of hypoglycemia (challenges 1 and 2 only), higher blood glucose nadir and reduced hypoglycemia exposure (area under the curve [AUC] of blood glucose <3.9 mmol/L) (*P* < .05 for all parameters), with a larger overall glucagon response in SSTR2a-treated rats (*P* < .0001 for glucagon parameters) compared to vehicle-treated rats. Furthermore, antecedent hypoglycemia affected glycemic parameters, but not the glucagon response in SSTR2a-treated rats, as blood glucose nadirs were lower (*P* < .01) and blood glucose AUC less than 3.9 mmol/L higher (*P* < .0001) in challenges 3 and 4 than in the first 2 challenges (Table 1**)**. In other words, the level of hypoglycemia progressively increased in both groups with repeated exposures.

**Table 1.** Blood glucose responses to insulin-induced hypoglycemic challenges 1 to 4 (study B)

Metric	Challenge 1		Challenge 2		Challenge 3		Challenge 4		Effect statistical significance	
	Vehicle	SSTR2a	Vehicle	SSTR2a	Vehicle	SSTR2a	Vehicle	SSTR2a	Treatment	Antecedent hypoglycemia
Group size, N	9	10	9	9	8	6	8	6		
Baseline blood glucose, mmol/L	20.4 ± 4.0	23.1 ± 3.9	21.9 ± 3.1	22.1 ± 4.4	21.6 ± 5.3	21.5 ± 3.8	18.8 ± 2.9	22.4 ± 5.7	NS	NS
Terminal blood glucose, mmol/L	3 ± 0.6	3.3 ± 1.3	2.6 ± 0.6	3.3 ± 1.4	2.4 ± 0.6	2.2 ± 0.7	2.3 ± 0.7	2.5 ± 0.9	NS	NS
Blood glucose nadir, mmol/L	2.6 ± 0.5	3.3 ± 1.0	2.5 ± 0.5	3.3 ± 0.8	2.4 ± 0.4	2.2 ± 0.6	2.1 ± 0.6	2.4 ± 1.0	*P* < .05	*P* < .01
Blood glucose AUC <3.9 mmol/L	36.2 ± 22.5	14.8 ± 21.1	35.9 ± 12.2	10.2 ± 16.1	57.8 ± 27.6	58.8 ± 44.7	83.1 ± 49.1	60.5 ± 35.1	*P* < .05	*P* < .0001
Median time to hypoglycemia onset, min	60	105	75	105	60	53	45	60	Challenge 1: *P* = .01 Challenge 2: *P* = .004 Challenge 3: NS Challenge 4: NS	
% Hypoglycemia	100%	70%	100	67%	100%	100%	100	83%		
Fold-increase in glucagon	6.4 ± 7.0	15.9 ± 8.0	7.4 ± 8.7	26.9 ± 16.2	12.0 ± 8.1	12.4 ± 9.0	13.6 ± 12.5	14.4 ± 8.8	*P* < .0001	NS
Tmax glucagon, min	63 ± 46	39 ± 45	103 ± 22	10 ± 30	83 ± 35	30 ± 50	94 ± 40	30 ± 38	*P* < .0001	NS

Blood glucose metrics comprising of baseline (at t = 0), terminal (at t = 120), nadir glucose levels, time to hypoglycemia, and percentage of animals that became hypoglycemic is recorded for all 4 hypoglycemic challenges. Data are represented at mean ± SD, and statistically significant differences are denoted by *P* values less than .05. NS indicates *P* less than .05.

Abbreviations: AUC, area under the curve; NS, not significant; SSTR2a, somatostatin receptor antagonist; Tmax, maximal glucagon response.

In hypoglycemic challenge 1 (Fig. 4A), the rate and extent of hypoglycemia was statistically significantly lower in animals administered SSTR2a compared to vehicle. Median onset of hypoglycemia was 105 vs 60 minutes, and hypoglycemia incidence was reduced by 30% with SSTR2a compared to controls (*P* = .01). Total hypoglycemic exposure AUC for blood glucose less than 3.9 mmol/L mean values were 36.2 vs 14.8 mmol/L × min (Fig. 4F). Similar reductions in hypoglycemia exposure were observed in challenge 2 (Fig. 4B): a 30-minute delay in hypoglycemia onset (median onset time of 75 vs 105 minutes) and a 33% reduction in hypoglycemia incidence (*P* = .004), and blood glucose AUC less than 3.9 mmol/L of 35.9 vs 10.2 mmol/L × min in the vehicle compared to SSTR2a groups. However, no statistically significant differences were observed in challenges 3 and 4, although a numerical reduction in the incidence rate of hypoglycemia (by 17%) was observed in challenge 4 with SSTR2a compared to vehicle (Fig. 4C and 4D). Blood glucose nadirs in each challenge also declined with antecedent hypoglycemia, while overall hypoglycemia exposure (blood glucose AUC <3.9 mmol/L × min) increased (see Table 1).

#### More responsive glucagon and C-peptide with somatostatin receptor antagonist administration

**Figure 5.**
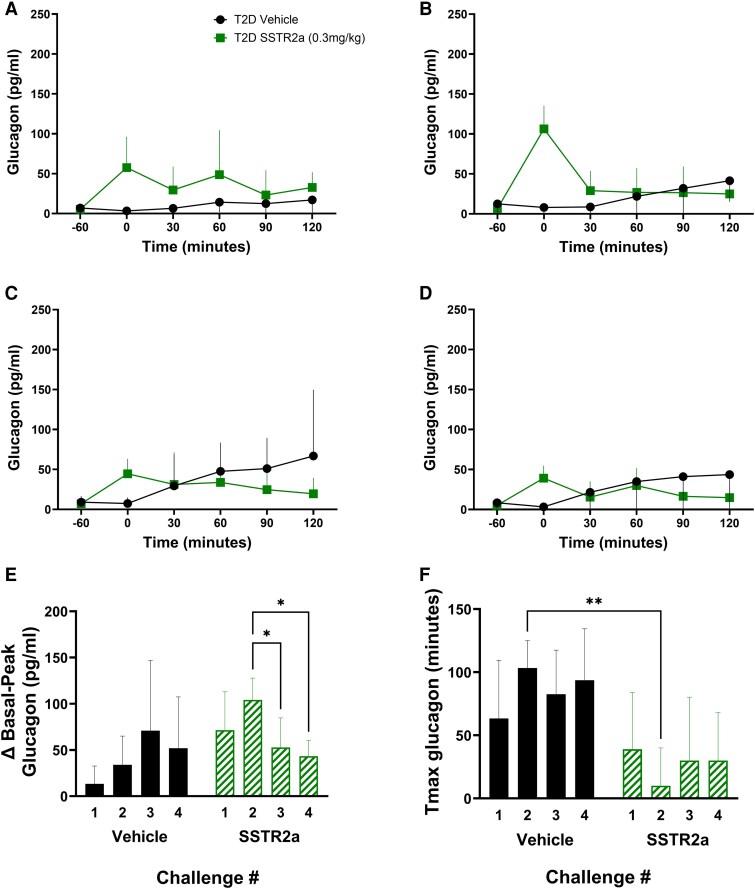
Glucagon responses to drug or vehicle administration over 4 successive insulin-induced hypoglycemic challenges. Glucagon responses to drug or vehicle over the 4 hypoglycemic challenges are denoted in panels A to D, respectively. The difference between baseline and peak glucagon (Δ basal – peak) is shown in E and the time to peak glucagon for each challenge is denoted in F (Tmax). Drug or vehicle was administered at t = −60 minutes (baseline) and insulin was given at t = 0 minutes. A to D, Data are presented as mean ± SD for line graphs. For E and F, vehicle-treated groups are indicated in black circles, while somatostatin receptor antagonist (SSTR2a) groups are indicated in green squares. Statistical significance is denoted by *P less than .05 and **P less than .01.

For all challenges, mean baseline glucagon values were in the range of 5 to 13 pg/mL, with no differences between groups or changes over subsequent challenge days. In all challenges, glucagon levels during hypoglycemia were higher with SSTR2a administration compared to controls. Glucagon levels rose early in the challenge period in response to SSTR2a administration (Tmax values, time at which the peak glucagon was observed, were <40 minutes for all SSTR2a rats in all challenges) and levels remained elevated for 60 to 120 minutes (Fig. 5A-5D). In the vehicle group, no glucagon response was observed in challenge 1 (Fig. 5E), while in later challenges a glucagon response with slower onset (compared to the SSTR2a group) was observed in most rats, with Tmax values greater than 90 minutes (Fig. 5F). The glucagon response, expressed in terms of fold-change from basal (baseline, prior to drug administration) to peak values (highest glucagon level), was significantly greater in the SSTR2a group in the first 2 challenges, but not in the final 2 challenges (see Table 1; Fig. 5E). The fold-change in glucagon levels following SSTR2a administration during challenges 3 and 4 were similar to those observed in the first 2 challenges. However, in challenges 3 and 4, glucagon also rose to a similar extent in the vehicle-treated rats, but was increased only toward the end of the hypoglycemic challenge (Tmax; Fig. 5F).

**Figure 6.**
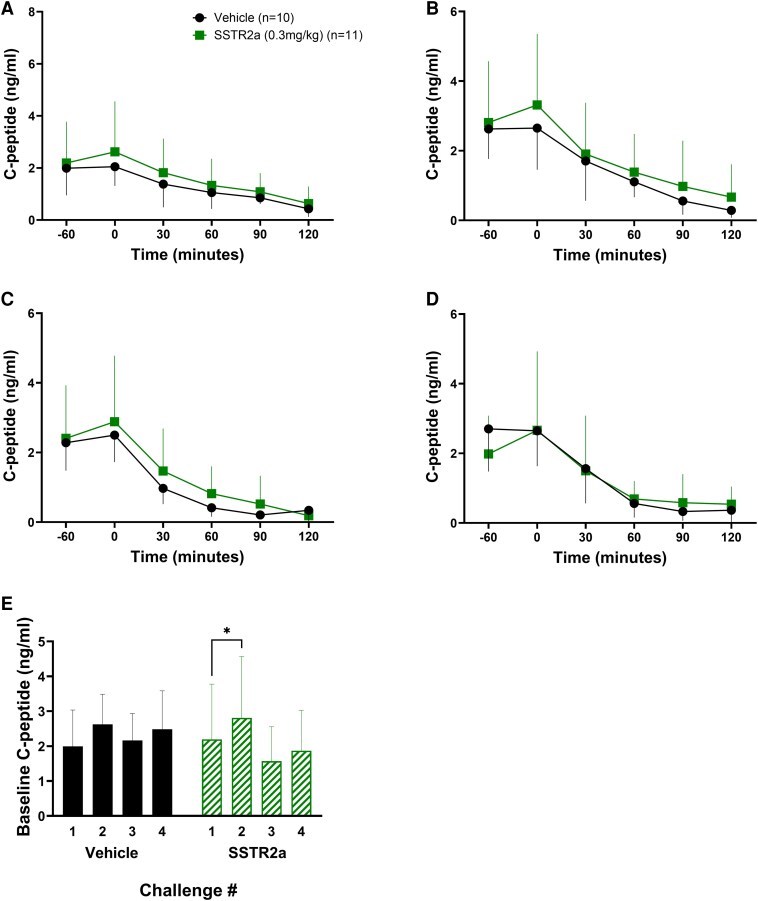
C-peptide responses to 4 successive insulin-induced hypoglycemic challenges. C-peptide measurements were recorded in challenges 1 to 4 (A-D, respectively). Baseline (t = −60) levels prior to drug or insulin administration are presented in panel E. Somatostatin receptor antagonist (SSTR2a)-treated animals are indicated by green squares and vehicle-treated animals are represented by black circles. Drug or vehicle was administered at t = −60 minutes and insulin was given at t = 0 minutes. A to D, Data are presented as mean ± SD for line graphs. For E, vehicle-treated groups are indicated in black circles while SSTR2a groups are indicated in green squares. Statistical significance is denoted by *P less than .05.

In all 4 challenges, C-peptide levels were similar at baseline in both groups (1.6-2.8 ng/mL), and declined, independent of SSTR2a, to very low levels within 60 minutes of insulin administration (Fig. 6A-6D). During all 4 challenges, in SSTR2a-treated rats, C-peptide levels rose by approximately 0.5 ng/mL from baseline at t = 0 (prior to insulin dosing), but not in vehicle-treated rats (*P* = .02). Across all challenges, the mean C-peptide concentrations at t = 0 in SSTR2a- and vehicle-treated rats were 3.25 ± 1.9 ng/mL and 2.8 ± 1.6 ng/mL at baseline, respectively (Fig. 6E).

## Discussion

The goal of this work was to determine the possible long-term effects on overall glycemia and hormone responses to recurrent insulin-induced hypoglycemia with daily exposure to SSTR2a, in the context of insulin dosing (a) for glycemic management (ie, daily maintenance doses) or (b) for repeated intentional induction of hypoglycemia, in male rats with T2D. Compared to vehicle-treated controls, daily SSTR2a administration did not alter food intake, body weight, or basal/bolus maintenance insulin needs to treat hyperglycemia, but did result in a lower HbA_1c_ level and fasting glycemia than controls after 8 daily doses, and increased glucagon response during repeated insulin-induced hypoglycemia. These findings demonstrate the potential for SSTR2a administration to be used to increase glucagon secretion in the context of glucagon counterregulatory failure, which can lead to increased risk of hypoglycemia.

In study A, glucagon levels were affected both in a time- and treatment-dependent fashion. Baseline glucagon (before SSTR2a/vehicle dose) declined from day 1 to day 8, independent of treatment (both in the SSTR2a and vehicle groups), while glucagon levels increased only following SSTR2a treatment, but not with vehicle treatment. Because of the overall decline in glucagon over time, including the baseline and treatment effect, the durability of the SSTR2a effect was assessed in terms of fold-increase in glucagon with SSTR2a, which was consistent over time (∼23-fold), supporting the hypothesis that SSTR2 antagonism will elicit the same fold-increase from baseline with each daily dose. The 10-fold higher SSTR2a dose used in study A relative to study B was selected to assess if prolonged exposure to SSTR2a at a relatively high level would affect the endogenous capacity for glucagon secretion. The increased glucagon response with SSTR2a was also observed during hypoglycemia (study B) at a 10-fold lower dose than was used in study A, and no decline in baseline glucagon occurred over time in study B. A sustained effect with repeated SSTR2a dosing has also been reported in a study to assess tachyphylaxis in nondiabetic Sprague-Dawley rats (21). Taken together, these results indicate that the effect of SSTR2a administration on increased glucagon response is largely maintained under conditions of repeated dosing.

Repeat hypoglycemia challenges resulted in significant antecedent hypoglycemia effects, including faster onset and greater depth of hypoglycemia in both groups. Over the course of the repeated challenges, overall glycemia (a reduction of baseline hyperglycemia levels and number of rats achieving euglycemia—blood glucose <10.0 mmol/L) also improved in both groups over time. Coinciding with the improvements in overall glycemia, the vehicle group also exhibited a small but noticeable increase in peak glucagon response with each successive hypoglycemic challenge. However, the peak glucagon response was delayed in vehicle-treated rats relative to the SSTR2a group, occurring only toward the end of the challenge period and did not prevent hypoglycemia onset. In contrast, mean peak glucagon response occurred in response to dosing in the SSTR2a group, and remained elevated during hypoglycemia. It is important to acknowledge that SSTR2a treatment resulted in peak glucagon responses that were prior to bolus insulin challenge in each of the 4 experimental settings. This suggests that drug dosing may not elicit a glucagon response only as hypoglycemia develops, but also may raise it, at least transiently, even in the absence of hypoglycemia. Findings of blunted or delayed counterregulation are consistent with other reports of repeated hypoglycemia exposure (12, 38, 39), even in the context of more intensive glycemic control (13). Therefore, detection of SSTR2a-mediated effectiveness on hypoglycemia prevention could have been overpowered by the blunted counterregulatory effects caused by antecedent hypoglycemia, and improvements in overall glycemia in later challenges (3 and 4).

Improvements in overall glycemia over time were more pronounced in the SSTR2a group in study B, which had a significant drop in baseline glycemia over the 4 hypoglycemic challenge days (Fig. 4E), with more than double the animals being euglycemic at baseline compared to the vehicle group. Moreover, time to hypoglycemia decreased in both groups over challenges 1 to 4, and the decrease was greater in the SSTR2a-treated group by approximately 14 minutes (see Table 1). These findings suggest that drug efficacy was lost by the fourth hypoglycemic challenge, although there was still a nonsignificant delay in hypoglycemia onset by approximately 15 minutes in the drug-treated animals as compared to those treated with vehicle. Thus, it appears that antecedent hypoglycemia further deteriorates glucagon counterregulation, with or without drug treatment.

Unexpectedly, we observed a lower HbA_1c_ level in the SSTR2a-treated group, relative to controls at the end of the study. It is possible, therefore, that daily SSTR2a administration may be associated with glycemic improvement over time (rather than a deterioration), perhaps because of pulsatile glucagon secretion, which can stimulate insulin secretion (40, 41) and/or enhance hepatic insulin sensitivity (42).

Although HbA_1c_ typically reflects the glycemic level over a period of months, 2-week periods (in rats and humans) are sufficient to detect changes in glycemic control in diabetes by this metric (43-46). Different rodent studies have shown various ranges of HbA_1c_ ranging from 6% to 11%, as reviewed elsewhere (47), which have similar thresholds to humans for prediabetes at 5.7% to 6.4% (48), while HbA_1c_ in the range of 5.0% to 5.5% is also associated with a 2-fold higher risk of diabetes compared to levels less than 5.0% (49).

A potential mechanism by which SSTR2a may help to improve glycemia in T2D rats is by acting on functional β cells in these animals. In addition to improved glycemia over time, we observed higher C-peptide levels 1 hour following SSTR2a administration in study B, before levels declined in response to hypoglycemia. In addition to suppressing glucagon counterregulation via SSTR2a receptors on α cells, endogenous SST suppresses insulin secretion via SSTR5 on β cells. SST present as both SST14 (pancreatic) and SST28 (gut derived) (50) can act differentially in the islet. SST14 more potently inhibits α-cell glucagon secretion whereas SST28 strongly inhibits insulin secretion from β cells (51), normally in hyperglycemic conditions (52). Because SST can be elevated in diabetes (19), inhibition of insulin secretion can occur via SST acting both on SSTR5 (the dominant β-cell receptor subtype) but also SSTR2, which is present but with 10-fold lower binding capacity for SST28 (53). It is unclear if SSTR2a could potentially influence β-cell function in health and/or diabetes via effecting endogenous SST binding to either receptor 2 or 5 on the β cell.

Previous studies have implicated the involvement of SST receptor antagonism on overall glycemic improvement, and these improvements were attributed to antagonism of SSTR5 and only minimal effects were noted with antagonism to SSTR2. However, these experiments were conducted in nondiabetic mice and involved a single (not daily) administration of SSTR2a and SSTR5a in studies conducted over a short (2-hour) duration (54). In contrast, this study evaluated effects over repeated daily doses during which changes could be observed with time. Other studies involved sustained delivery of SSTR2a in rats with T1D using microneedle technology patches. However, these studies were also of short (2.5-hour) duration (55). It is possible that some changes in glycemia due to repeat dosing of SSTR2a can only be observed with a daily dosing schedule over several days, in rats undergoing glycemic management with daily insulin, which may itself have also contributed to glycemic improvements.

A strength of this study is its design to investigate repeated daily SSTR2a dosing not only under hyperglycemic conditions but also under conditions of repeated hypoglycemia, enabling assessment of SSTR2a over time and under varying glycemic states of relevance to diabetes management. This study also evaluated SSTR2a in conjunction with small and large insulin dosages, covering a 10-fold range up to 12 U/kg, sufficient to induce hypoglycemia in all vehicle-treated (ie, control) rats. This wide range of parameters evaluated enabled observation of the effects of SSTR2a expected based on its mechanism of action, but also of improved overall glycemia, which was not expected. However, as the glycemic improvements observed in study B were an unexpected finding, additional measures that would support this finding were not planned, including assessment of HbA_1c_ at baseline, and glucose and insulin tolerance tests. Future studies aiming to more robustly demonstrate improvements in overall glycemia by SSTR2a administration could be designed with more comprehensive recording of baseline glycemic parameters and evaluate changes without antecedent hypoglycemia and under different glycemic management strategies such as varying intensity of maintenance insulin dosing. These studies would prove valuable to assess the effect of repeated drug exposures (both with any long-acting formulations or sustained drug delivery and with pulsatile SSTR2a exposures) on overall glycemic improvements. Finally, additional parameters such as sex differences in glycemic and hormone responses were outside the scope of this study but should be evaluated in future assessments of prolonged exposure to SSTR2a.

It should be acknowledged that while glycemic and general metabolic effects of prolonged exposure to SSTR2a in a rodent model of T2D were the focus of this study, SSTR2 is also present on extrapancreatic tissues such as the kidney, brain, liver, and inflammatory cells (56, 57). Additionally, it can also inhibit growth hormone and thyrotropin in healthy rat and human pituitary cells (58). Future studies should explore the possibility that this and other SSTR2 antagonists may alter hormone secretions in these tissues in rodent models of diabetes.

In conclusion, the results of this study demonstrate that daily SSTR2a administration increases endogenous glucagon secretion, reducing overall hypoglycemia exposure, and that these effects may be limited in this model by factors including effects of antecedent hypoglycemia and due to overall improvement of glycemic status and glucagon counterregulatory capability of the rats over time. Furthermore, daily SSTR2a administration may have promoted improvement in normalization of blood glucose levels in this T2D model.

## Data Availability

Some or all datasets generated during and/or analyzed during the current study are not publicly available but may be available from the corresponding author on reasonable request.

## Abbreviations

AUC: area under the curve
HbA_1c_: glycated hemoglobin A_1c_
IP: intraperitoneal
SH: severe hypoglycemia
SST: somatostatin
SSTR2a: somatostatin receptor antagonist
STZ: streptozotocin
T1D: type 1 diabetes
T2D: type 2 diabetes
Tmax: maximal glucagon response
